# Environmentally Determined Differences in the Murine Lung Microbiota and Their Relation to Alveolar Architecture

**DOI:** 10.1371/journal.pone.0113466

**Published:** 2014-12-03

**Authors:** Yeojun Yun, Girish Srinivas, Sven Kuenzel, Miriam Linnenbrink, Safa Alnahas, Kenneth D. Bruce, Ulrich Steinhoff, John F. Baines, Ulrich E. Schaible

**Affiliations:** 1 Research Center Borstel, Cellular Microbiology Group, Department of Molecular Infection Biology, Borstel, Germany; 2 MPI for Evolutionary Biology, Plön, Germany; 3 Department of Dermatology, University of Lübeck, Lübeck, Germany; 4 Evolutionary Genomics, Institute for Experimental Medicine, Christian-Albrechts-University, Kiel, Germany; 5 Institute for Medical Microbiology and Hospital Hygiene, Philipps University Marburg, Marburg, Germany; 6 Institute of Pharmaceutical Science, King's College London, London, United Kingdom; University of Pittsburgh, United States of America

## Abstract

Commensal bacteria control the micro-ecology of metazoan epithelial surfaces with pivotal effect on tissue homeostasis and host defense. In contrast to the upper respiratory tract, the lower respiratory tract of healthy individuals has largely been considered free of microorganisms. To understand airway micro-ecology we studied microbiota of sterilely excised lungs from mice of different origin including outbred wild mice caught in the natural environment or kept under non-specific-pathogen-free (SPF) conditions as well as inbred mice maintained in non-SPF, SPF or germ-free (GF) facilities. High-throughput pyrosequencing of reverse transcribed 16S rRNA revealed metabolically active murine lung microbiota in all but GF mice. The overall composition across samples was similar at the phylum and family level. However, species richness was significantly different between lung microbiota from SPF and non-SPF mice. Non-cultivatable Betaproteobacteria such as *Ralstonia* spp. made up the major constituents and were also confirmed by 16S rRNA gene cloning analysis. Additionally, Pasteurellaceae, Enterobacteria and Firmicutes were isolated from lungs of non-SPF mice. Bacterial communities were detectable by fluorescent *in situ* hybridization (FISH) at alveolar epithelia in the absence of inflammation. Notably, higher bacterial abundance in non-SPF mice correlated with more and smaller size alveolae, which was corroborated by transplanting *Lactobacillus* spp. lung isolates into GF mice. Our data indicate a common microbial composition of murine lungs, which is diversified through different environmental conditions and affects lung architecture. Identification of the microbiota of murine lungs will pave the path to study their influence on pulmonary immunity to infection and allergens using mouse models.

## Introduction

Human-associated microbiota have been characterized at many body sites including the gut [1] and epithelial surfaces including the skin [2], vagina [3], oral cavity [4] and upper respiratory tract (RT) [5]. Of these the gut, oral cavity and upper respiratory tract are particularly heavily colonized by a diverse range of bacterial species in healthy individuals [6]. There is consensus that the lower airways can be heavily colonized by microbes in clinical scenarios such as cystic fibrosis (CF) [7], asthma [8], [9] and chronic obstructive lung disease (COPD) [8], [10]. In contrast, the lower airways of healthy individuals have been considered by some authors as sterile [11]. However, evidence for the presence of microbes in the healthy lung in porcine and murine models has been obtained recently [12], [13]. Similarly, the presence of “*low levels of bacterial sequences largely indistinguishable from upper respiratory flora*” in the healthy human lung has also been reported [14]. Despite the careful precautions taken in the previous study, a recurring concern is over the potential for contamination arising from the passage of lower respiratory tract samples through the heavily colonized regions of the upper airways and oral cavity.

Excising lung material directly from dead mice offers one means of avoiding contamination from species in the oral cavity and upper respiratory tract. As such, this allows the identification of bacterial species present in the lower airways at the time of sampling, as well as species detectable in lower airways of different mouse populations. In the present study, this approach was used to identify bacterial species in lungs from mice, which were either out- or inbred and genetically defined and kept under different environmental conditions, i.e. caught in the wild, raised conventionally (non-SPF), under SPF – or under germfree conditions (see Materials and Methods for details).

To identify and compare the bacterial compositions in lower airway tissues of excised murine lungs, microbiological culture as well as culture-independent 16S rRNA gene sequencing was performed [15]. Additionally, identified microbial species were further characterized by their colonization potential.

## Materials and Methods

### Mice

Germ-free (GF) C57BL/6 mice were provided by the University of Marburg. GF animals were kept in plastic isolators (Metall and Plastik, Germany) with autoclaved food, bedding and water. Sterility of animals was checked biweekly by culturing feces in thioglycollate medium under aerobic and anaerobic conditions for at least ten days. All handling procedures for GF mice, including infection experiments, were conducted in a laminar flow hood under sterile conditions. SPF raised C57BL/6 mice were purchased from Charles River (Germany). They were kept for a short term under SPF conditions at the animal facility of the Research Center Borstel in individually ventilated cages till used. SPF mice were nourished with sterile chow and regularly tested microbiologically. Non-SPF kept C57BL/6 mice originally also purchased from Charles River (Germany) (termed “non-SPF C57BL/6 mice”) and wild-derived mice originating from natural areas in France and Germany were bred in a separate facility maintained by the Max Planck Institute for Evolutionary Biology in Plön, Germany. For organ extraction, mice were euthanized by CO_2_ inhalation to avoid mechanical disruption of the lung and respiratory tract tissue and thus, potential contamination from tracheal microbiota. The procedure was approved by the Ethics Committee for Animal Experiments of the Ministry for Agriculture, Environment, and Rural Areas of the State of Schleswig-Holstein, Germany, i.e. Kommission für Tierversuche/Ethik-Kommission des Landes Schleswig-Holstein (V 312-72241.123-34 according to TierSchG §6 Abs1 Nr.4). All wild-derived mice belong to the *Mus musculus domesticus* subspecies and were at least one generation removed from their free-living progenitors (*i.e*. were born in the lab). Each non-SPF C57BL/6 and wild-derived mouse was housed in separated racks with environmental air and fed the same standard chow.

In addition, wild *M. m. domesticus* were caught in the Massif Central region of France (termed “wild-caught mice”), sacrificed with CO_2_ and dissected directly in the field as described in Linnenbrink et al [16]. Utensils were washed in 70% ethanol and flame-sterilized before each individual dissection, and a separate set of instruments was used to remove lungs after opening the thoracic cavity. Table 1 displays the origin and number of mice that were analyzed in individual experiments.

**Table 1:** The number of mice used in each experiment.

	GF C57BL6	SPF C57BL6	Non-SPF C57BL6	Wild derived	Wild caught
**total mice**	6	6	6	12	15
**Culture**	ND*	2	6	12	ND
**Cloning**	ND	2	6	12	ND
**454 pyrosequencing**	ND	3	4	8	15
**FISH & Histology**	6	6	4	6	ND

*Not determined.

### DNA/RNA Extraction and cDNA preparation

For the culture-independent analyses, both RNA and DNA were simultaneously extracted from lung tissue samples frozen in liquid nitrogen or fixed in RNAlater (Ambion) using the AllPrep DNA/RNA Mini Kit following the manufacturer's instructions (Qiagen). Isolated RNA was used as template to generate cDNA employing the 926r primer and SuperScript III Reverse Transcriptase (Invitrogen) following the manufacturer's instructions. 16S rRNA derived cDNAs were used as templates for 16S rRNA gene pyrosequencing and cloning procedure.

### 16S rRNA gene pyrosequencing sequencing and analysis

The primer pair 27F-338R flanking the V1 and V2 hypervariable regions of the bacterial 16S rRNA gene was used for PCR and barcoded pyrosequencing on the 454 GS-FLX with Titanium sequencing chemistry as described by Rausch et al. [17]. The forward primer and barcode sequences were identified by a Perl script using a Smith-Waterman alignment allowing no insertions or deletions. Sequences were required to have a minimum length of 290 nucleotides and quality score of ≥20. Chimeric sequences were removed using ChimeraSlayer (http://microbiomeutil.sourceforge.net/). RDP Multi-Classifier version 1.0 was used to assign taxonomy with a minimum confidence score of 0.80. Clustering of sequences into operational taxonomic units (OTUs) at the species-level similarity threshold (97%) and alpha diversity estimates were performed using QIIME version 1.7.0 [18]. UCLUST reference based OTU picking was implemented against a representative dataset (“97_otus.fasta” available from Greengenes database release 13_5; http://greengenes.secondgenome.com/downloads/ database/13_5) to define Operational Taxonomic Unit (OTUs) at 97% sequence identity and discard singleton OTUs. Reads that did not match the collection of reference sequences were subsequently clustered as *de novo* clusters. We used the Chimeraslayer algorithm using a reference non-chimeric database (http://drive5.com/uchime/gold.fa) for detecting Chimeric sequences. The raw sequence counts for samples from the 454 data ranged from 1178 to 9347. All samples were rarefied to 1000 sequences and the downstream analyses were performed on normalized counts. Beta diversity analyses were performed using the Vegan R package [19] including the taxon (OTU)-based Bray-Curtis and Jaccard distances as well as the phylogenetic-based unweighted and weighted UniFrac distances [20]. Alpha and beta diversity analyses were performed with a normalized sequence number of 1000 per individual. The entire dataset associated with this study was submitted to the European Nucleotide Archive under the study accession number PRJEB7007 (http://www.ebi.ac.uk/ena/data/view/PRJEB7007).

### Polymerase chain reaction (PCR) and Cloning procedures

cDNA template generated from each GF, SPF, non-SPF C57BL/6 and wild-derived mouse lung was amplified by 16S rRNA gene PCR primer set: 8f700 (5′-AGA GTT TGA TCC TGG CTC AG-3′) and 926r (5′-CCG TCA ATT CAT TTG AGT TT-3′) [7]. For the quality control of cDNA, *Ralstonia*-specific PCR was done using the primer set: Rp-F (5′-ATG ATC TAG CTT GCT AGA TTG AT-3′) and Rp-R (5′-ACT GAT CGT CGC CTT GGT G-3′) [21]. PCR reactions were perfomed by an initial denaturation step at 95°C for 5 min followed by 40 cycles of denaturation at 95°C for 30 s, primer annealing at 60°C for 30 s and primer extension at 72°C for 45 s, with final extension step at 72°C for 5 min.

Cloning of 16S rRNA PCR-amplified DNA was performed with the TOPO TA cloning kit (Invitrogen) according to the manufacturer's instructions. Transformation was performed with competent *Escherichia coli* TOP10 cells provided by the manufacturer. The transformed cells were then plated onto LB agar plates supplemented with Ampicillin (100 µg/ml), and the plates were incubated overnight at 37°C. Correct sizes of the inserts were determined in a colony PCR with an M13 forward and reverse primer. Prior to sequencing of the fragments, the PCR-amplified 16S rRNA gene regions were purified with the GeneJET PCR Purification Kit (Fermentas).

### Culture collection from mouse lung

The lungs of all mice, apart from those sampled directly in the wild, were collected under a clean bench to avoid contamination of extraneous components. The trachea was removed before lungs were excised carefully without any upper respiratory parts. One lobe of lung was transferred to a Dispomix (gentleMACS Dissociator, Miltenyi Biotec) disposable homogenizer with 500 uL sterile phosphate buffered saline (PBS). The homogenization step with the Dispomix was performed at 500 *g* for 20 seconds. After homogenization the centrifuged sample was gently mixed and transferred to different bacterial culture media: LB plate/broth, BHI plate/broth (aerobic/anaerobic), anaerobic blood agar plate (BAP), anaerobic Fluid thioglycolate medium (FTG), and 5% CO_2_ blood agar (BAP)/chocolate agar plate (CH). To assess the number of cultivatable bacteria in mouse lungs, colonies were counted from each BHI and BAP plate. Pure colonies were isolated and subcultured for further analysis.

### DNA extraction and identification of murine lung isolates

DNA was extracted from individual bacterial isolates by the Phenol/chloroform/isoamyl (PCI) method after treatment with lysozyme and proteinase K and used as template for 16S rRNA gene PCR. Two universal bacterial primers were used: 8f700 (5′-AGA GTT TGA TCC TGG CTC AG-3′) and 926r (5′-CCG TCA ATT CAT TTG AGT TT-3′) [7].

After sequencing, 11 culture collections of representative taxa were deposited in Leibniz-Institut DSMZ (Deutsche Sammlung von Mikroorganismen und Zellkulturen GmbH, which are DSM26538 (*Staphylococcus xylosus*, G2B1), DSM26539 (*Pasteurella pneumotropica*, 3F1B2), DSM26540 (*Actinobacillus muris*, G2CH1), DSM26541 (*Enterococcus gallinarum*, 3B1CH1), DSM26543 (*Bacillus lichenifomis*, 3G1B1), DSM26544 (*Paenibacillus sp.* G3B2), DSM26546 (*Lactobacillus sp.* 3B2BAP1), DSM26547 (*Corynebacterium sp.* G1CH1), DSM26548 (*Lactobacillus sp.* 3F2BAP4), DSM26549 (*Escherichia coli*, 3B3BAP1), and DSM26621 (*Streptococcus danieliae* 3G1BAP1).

### Sequencing analysis

16S rRNA gene sequences from bacterial isolates and cloning were sequenced by the Sanger method (Seqlab, Germany). Sequences were aligned using Sequence Match of the ribosomal database project (RDP, http://rdp.cme.msu.edu/) and compared to NCBI BLAST results for the percentage of sequence identity. The phylogenetic trees were conducted in MEGA4 software determined by the Neighbor-Joining method. All positions containing alignment gaps and missing data were eliminated only in pairwise sequence comparisons. Branches corresponding to partitions reproduced in less than 50% bootstrap replicates were collapsed. The percentage of replicate trees in which the associated taxa clustered together in the bootstrap test (1000 replicates) was shown next to the branches. The sequences used in Figure S1 in File S1 were stored as GenBank accession KJ729555-KJ729591.

### Organs for histology and fluorescent *in situ* hybridization (FISH)

GF, SPF and non-SPF C57BL/6 mice between 24 and 25 weeks of age were compared for pulmonary histological differences. The age of wild–derived mice varied between 25–45 weeks.

For histopathological and FISH analysis, lungs were sterilely removed. To avoid alterations and redistribution of lung microbiota, lungs were immediately fixed by 4% paraformaldehyde at 4°C for 24 hours. Organs were further processed for paraffin embedding.

### Histology

Consecutive sections of 8 µm taken at regular intervals were obtained using a microtome. Sections were stained with either Haematoxylin-Eosin (H&E) or periodic acid-Schiff (PAS) to reveal mucus production according to standard procedures.

In short, paraffin sections on slides were deparaffinized and rehydrated. For H&E staining, slides were stained with Mayer's Haematoxylin followed by Eosin after rinsing with tab water. For PAS staining, slides were oxidized in 0.5% periodic acid solution, and then placed in Schiff reagent followed by counterstain with Mayer's Haematoxylin. Both procedures were finalized by dehydration and mounting.

Morphometry was done by measuring the size of alveolae and counting the numbers per microscopical field using a 400X magnification. Data represent the average of 10 microscopical fields. The alveolar area (µm^2^) of 10 alveolae was measured using Cell^B^ version 3.3 (Olympus Soft Imaging Solution GmbH).

### FISH

Hybridization was performed using the probe EUB338 labeled with Cy3, whose sequence is complementary to the sequence of a region within the 16S rRNA that is common to all eubacteria as well as the mixture probe, EUB338 I, II, III. The complementary probe non-EUB338 labeled with Cy3 was used as control. Tissue sections were prepared using sterile microtome equipment and deparaffinized in coplin jars by two changes of xylene and dehydrated twice with 99.9% ethanol for 3 min at each step. Slides were air dried and a circle was drawn around the tissue section using a hydrophobic pen (Dako PAP pen; Glostrup, Denmark). The hybridization and washing steps were performed as previously reported [22]. Fixed samples were hybridized by the application of 10 ml of hybridization buffer containing 5 ng of each specific oligonucleotide probe at 45°C for 90 min. Stringent washing was performed by incubating the slide in washing buffer (20 mM Tris-HCl pH 7.6, 0.01% sodium dodecyl sulfate, 112 mM NaCl) at 48°C for 15 min. Finally, the slides were rinsed with water, stained with DAPI, air dried, and mounted in Citifluor (Citifluor Ltd., London, United Kingdom). Slides were examined using a Nikon fluorescent microscope equipped with a standard filter set (Nikon, Germany).

### Murine lung microbiota transplants

The following bacterial isolates from C57BL/6 mice raised under non-SPF conditions were used for colonization experiments: DSM26546 (*Lactobacillus sp.* 3B2BAP1) and DSM26548 (*Lactobacillus sp.* 3F2BAP4) were isolated by culture in Lactobacilli MRS medium (Difco) in a 5% CO_2_ atmosphere. Isolates were freshly cultured at 37°C overnight, and washed 3 times in PBS. 5×10^5^ CFU in 20 µL PBS were given intranasally into both nostrils of 10 weeks old GF mice. After 16 weeks, mouse lungs were isolated and analyzed for lung colonization by *Lactobacillus* spp. specific PCR: F(5′-AGCAGTAGGGAATCTTCCA-3′) and R(5′-CACCGCTACACATGGAG-3′). [23] Amplification program included an initial denaturation step at 95°C for 5 min followed by 30 cycles of denaturation at 95°C for 30 s, primer annealing at 60°C for 30 s and primer extension at 72°C for 45 s, with final extension step at 72°C for 5 min. Paraffin embedded lung sections were analyzed for histological alterations by H&E and PAS stain (see above).

### Statistical analysis

Statistical analyses were conducted using GraphPad Prism version 5.01 (GraphPad software Inc., USA). Statistical significance was determined by one-way analysis of variation (ANOVA) followed by Tukey's Multiple comparison test or two tailed Student's t-test followed by Mann Whitney test (*p<0.05, **p<0.01, ***p<0.001). Statistical analysis of alpha diversity was performed using the Wilcoxon rank sum test followed by a correction for multiple testing [24]. The multivariate ANOVA procedure implemented in analysis of dissimilarity (“*Adonis*”) (included in Vegan R package) was used to test the statistical significance of beta diversity measurements with respect to the origin of mice with 1000 per mutations.

## Results

### Murine lung microbiota by 454 pyrosequencing

In this study, nucleic acids were extracted directly from different mouse populations originating from the wild or from animal suppliers and held under GF, SPF or non-SPF conditions. Lungs of these mice were compared to those of outbred *Mus musculus domesticus* populations derived from the wild, either housed under non-SPF conditions or sampled directly in the natural environment. cDNA from RNA extracts was used as template for 454 pyrosequencing using the ‘926r’ 16S rRNA primer for reverse transcription, since direct 16S rRNA gene amplification by PCR from lung tissue DNA failed, most likely due to the low amount of bacterial nucleic acid template in the tissue samples [7]. From these cDNA templates, a total of 90,662 16S rRNA gene region sequences averaging 3,022, were generated per sample from 30 excised lungs.

**Figure 1:**
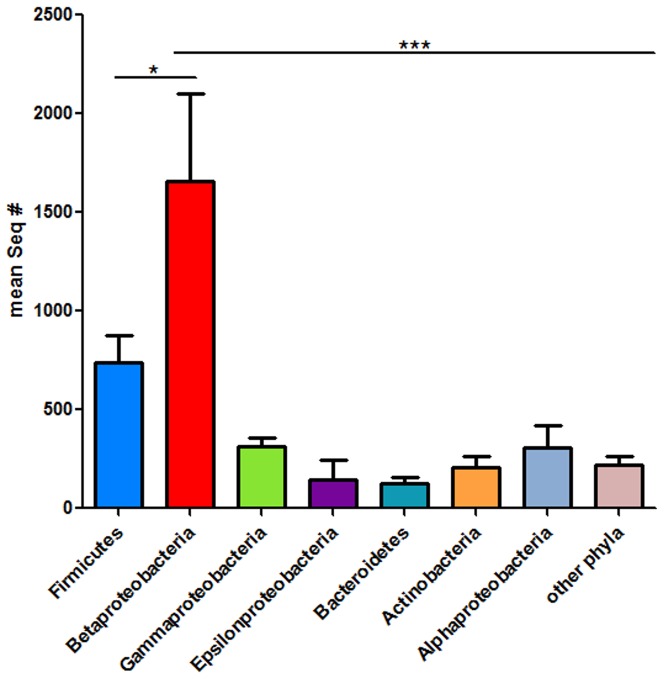
Bacterial 16S rRNA gene amplified by 454 pyrosequencing. Bar chart expressing the mean ± SEM of sequence numbers by 454 pyrosequencing. cDNA from 15 mouse lungs – 3 SPF, 4 non-SPF C57BL/6 and 8 wild-derived mice was amplified using primers specific for the 16S rRNA V2 region, and analyzed by 454 pyrosequencing. One-way analysis of variation (ANOVA) followed by Tukey's Multiple comparison test.

These results revealed distinct bacterial populations in murine lungs, with Betaproteobacteria dominating over Firmicutes, Gammaproteobacteria, Epsilonproteobacteria and Actinobacteria (Figure 1). Of note, Bacteroidetes and Alphaproteobacteria, which cannot be cultured easily, were identified in mouse lungs by 454 pyrosequencing in similar frequencies to Gammaproteobacteria (Figure 1).

To confirm and complement 454 pyrosequencing data, lung samples were additionally subjected to I) direct nucleic acid extraction for culture-independent 16S rRNA gene analysis by generating cDNA libraries using the ‘926r’ reverse primer for reverse transcription, and II) culture under aerobic, anaerobic or micro-aerophilic conditions using different growth media. We were able to amplify 16S rRNA gene from non-SPF as well as SPF, but not from GF mouse lungs. Cloned sequences of 16S rRNA genes were grouped upon phylogenetic analysis by the Neighbor-Joining method (shown by the numbers in red in Figure S1 in File S1). We identified Betaproteobacteria, primarily *Ralstonia* spp., as well as Firmicutes and Epsilonproteobacteria (*Helicobacter* spp.).

**Figure 2:**
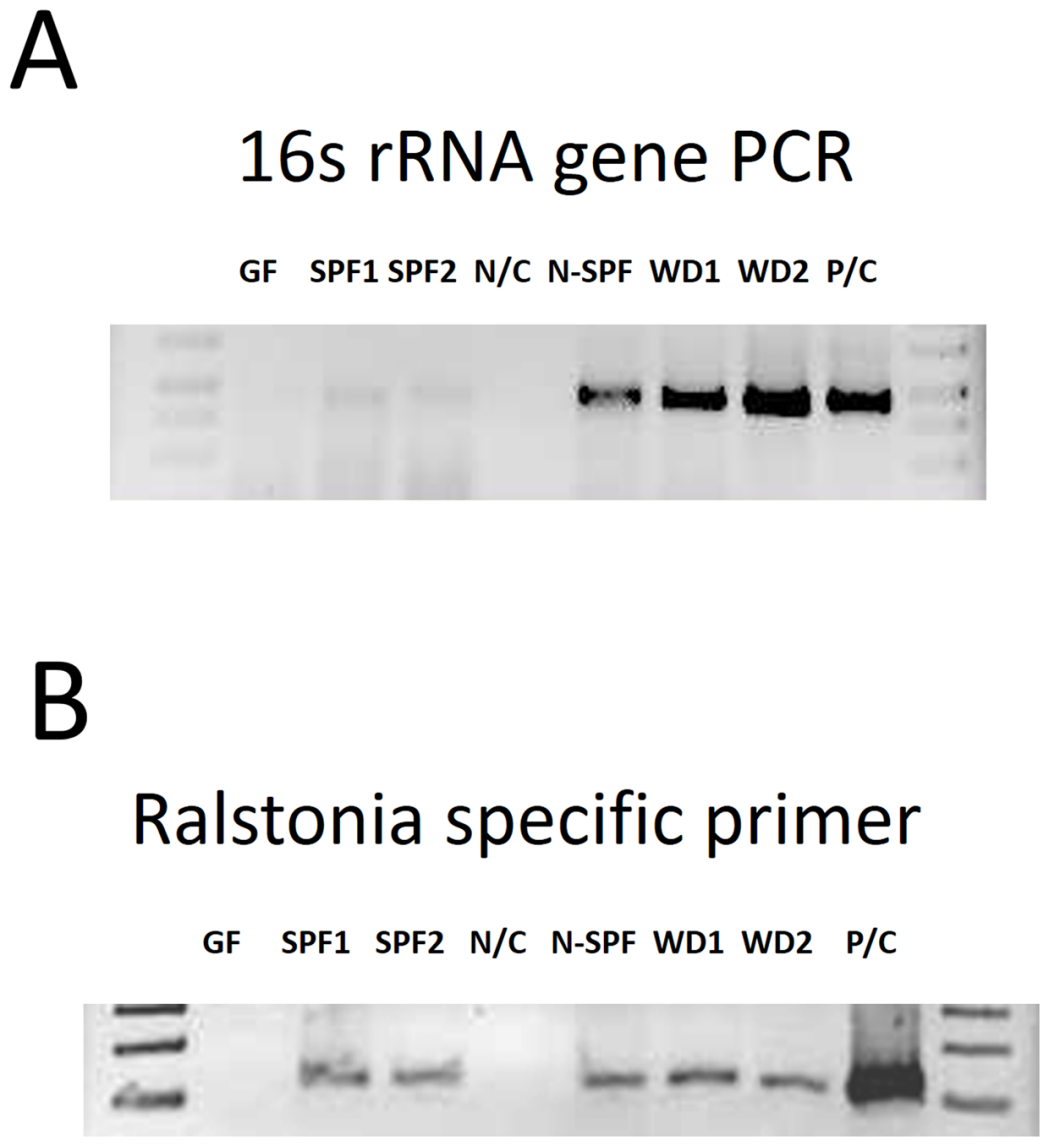
16S rRNA gene and *Ralstonia*-specific PCR. 16S rRNA gene PCR (A) and *Ralstonia*-specific PCR (B) (Abbreviations: GF; Germ Free mouse, SPF1 and SPF2; Specific Pathogen Free C57BL/6, N/C; negative control, N-SPF; non-SPF C57BL/6, WD1 and WD2; wild-derived mice, P/C; positive control, primers used are described in Materials and Methods).

Of note, *Ralstonia* spp. was also found in SPF mouse lungs otherwise relatively low in microbes. Since *Ralstonia* is a common laboratory contaminant, the possibility that its presence in SPF mouse lungs was due to contamination during the experimental procedure had to be excluded. Therefore quality control of the samples used for sequencing was performed through *Ralstonia*-specific PCR of the 16S rRNA gene (Figure 2A) revealing that the experimental procedure employed avoided bacterial contamination as GF mouse samples generated and processed simultaneously did not yield PCR products. The presence of *Ralstonia* spp. was further confirmed by specific PCR in the lungs from SPF as well as non-SPF, but not in GF mice, also demonstrating that *Ralstonia*-specific signals were not due to contamination during the experimental procedure (Figure 2B).

More importantly, we were able to isolate a total of 128 bacterial colonies from 5/6 non-SPF C57BL6 and 12/12 wild-derived mice but failed to isolate bacteria from lungs of all GF, SPF as well as 1/6 non-SPF C57BL/6 mice. Figure S2 in File S1 shows the differences in numbers and diversity of colonies isolated from lungs of individual mice from different environments. From these isolates, the 16S rRNA gene sequence-based tree (as indicated by the numbers in blue in Figure S1 in File S1) revealed predominantly of Firmicutes and Gammaproteobacteria. The most common genus, *Lactobacillus*, was found in 88.9% of mice (16 out of a total of 18 mice comprising 6 non-SPF and 12 wild-derived ones) as isolated by FTG under anaerobic culture. *P. pneumotropica* and *A. muris* were only isolated from wild-derived mice using either BAP or CH plates in 44.4% (8 of 18) and 33.3% (6 of 18) of the mice, respectively (Table 2). These results demonstrate that the murine lung contains cultivatable bacterial species, when mice live under environmental conditions, but not when they are kept in a SPF facility.

**Table 2:** The number or percent of mice carrying a particular cultivatable bacterial taxon, which is shown as ‘Genus or Species’ as well as ‘Family or Order’ level.

GENERA or SPECIES	Total (n = 18)	Non-SPF C57BL6 (n = 6)	Wild-derived (n = 12)
*P. pneumotropica*	8 (44.4%)	0 (0%)	8 (66.7%)
*A. muris*	6 (33.3%)	0 (0%)	6 (50%)
*Escherichia*	7 (38.9%)	2 (33.3%)	5 (41.7%)
*Streptococcus*	7 (38.9%)	3 (50.0%)	4 (33.3%)
*Lactobacillus*	16 (88.9%)	5 (83.3%)	11 (91.7%)
*Enterococcus*	2 (11.1%)	2 (33.3%)	0 (0%)
*Staphylococcus*	8 (44.4%)	1 (16.7%)	7 (58.3%)
*Bacillus*	4 (22.2%)	1 (16.7%)	3 (25.0%)
*Paenibacillus*	3 (16.7%)	1 (16.7%)	2 (16.7%)
*Corynebacterium*	1 (0.56%)	0 (0%)	1 (8.33%)
**FAMILY or ORDER**			
Pasteurellaceae	10 (55.6%)	0 (0%)	10 (83.3%)
Enterobacteriaceae	7 (38.9%)	2 (33.3%)	5 (41.7%)
Firmicutes-Lactobacillales	17 (94.4%)	5 (83.3%)	12 (100%)
Firmicutes-Bacillales	12 (66.7%)	3 (50.0%)	9 (75%)
Actinomycetales	1 (5.56%)	0 (0%)	1 (8.3%)

### Microbiota composition and diversity according to origin of mice

**Figure 3:**
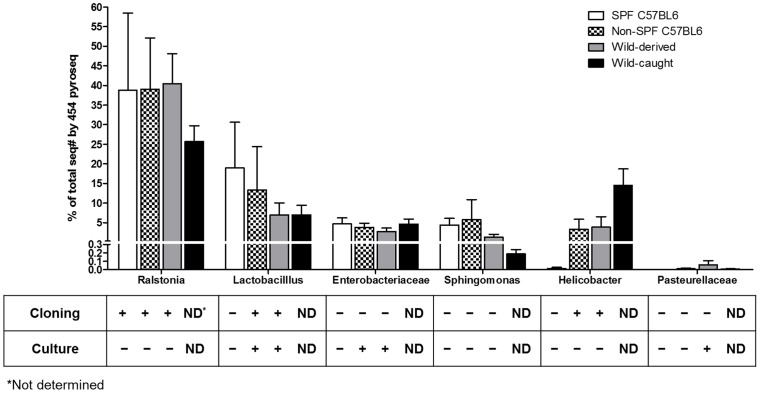
Composition of bacterial 16S rRNA gene sequences in each mouse group and comparison with cloning and culture results. The bar chart shows percent of total sequences of those assigned to individual genera or species as determined by 454 pyrosequencing and the corresponding results achieved by direct cloning and culture (“+” means detected, “−” not detected).

**Figure 4:**
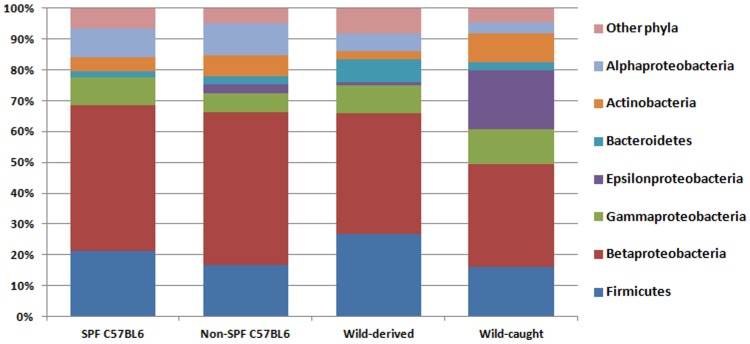
Bacterial compositions with respect to mouse origin. Evaluation of the distribution of bacterial phyla or classes from 454 sequencing data derived from each of the four groups of mice - 3 SPF, 4 non-SPF C57BL/6, 8 wild-derived, and 15 wild-caught mice.

The bacterial genera comprising the murine lung microbiota as identified by 454 16S rRNA pyrosequencing, direct 16S rRNA cloning and culture differed between the three methods of detection (Figure 3). In the cases of Enterobacteriaceae and Pasteurellaceae, the family names were used, i.e. *Escherichia* spp. and *Shigella* spp., and *Pasteurella* and *Actinobacillus*, respectively, as these genera are undistinguishable from their V2 region of the 16S rRNA gene. Only the genus *Lactobacillus* was identified by all three approaches. High throughput pyrosequencing resolved the discrepancy in species composition as detected by culture and the direct cloning approach. All mice, regardless whether they were of SPF or non-SPF origin, featured the same high ranking genera, i.e. *Ralstonia*, *Lactobacillus*, *Enterobacteriaceae*, and *Sphingomonas*. *Ralstonia* spp. ranked at the top of the list of all bacterial genera identified in mice caught in the wild, which was comparable in all mice studied independent of their origin. Interestingly, *Helicobacter* spp. (Epsilonproteobacteria) was abundant in wild-caught mice (Figure 3). At the phylum level, we found similar patterns in all mice analyzed, independent of their origin (Figure 4). In general, however, we observed considerable variability between mice of different origins at both the genus and family levels (Figure S3 in File S1). For example, *Bacteroides* (Firmicutes) was missing only from SPF lungs, but was abundant in mice caught in the wild (Figure S3B in File S1). *Enterobacteriaceae* (Gammaproteobacteria) were equally abundant in all mice analysed but not in wild-derived mice, which contained predominantly *Pasteurella* spp. (Figure S3E in File S1). It has to be noted that these comparisons were statistically not significant.

**Figure 5:**
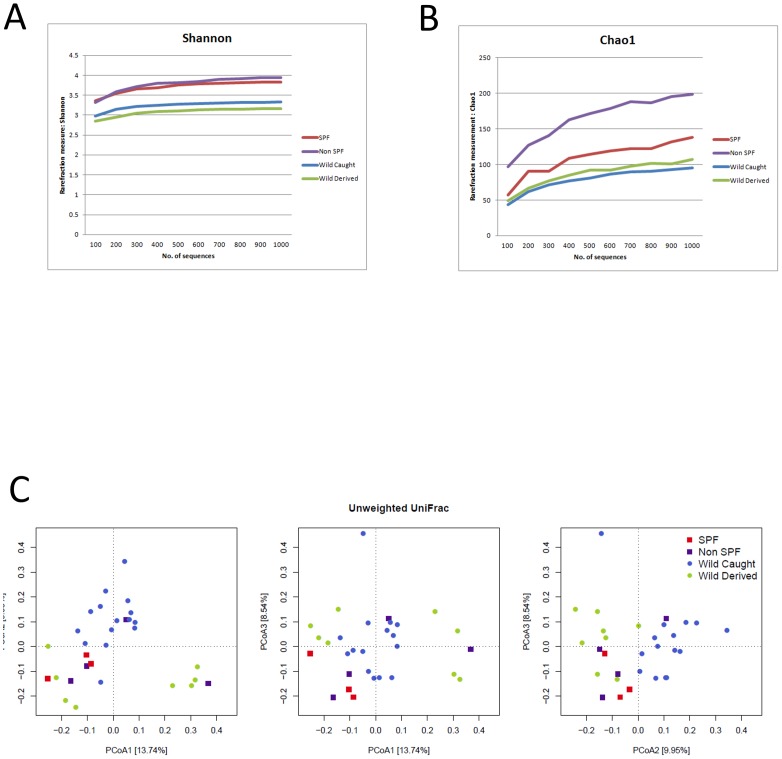
Bacterial diversity with respect to mouse origin. Rarefaction analysis of 16S rRNA gene sequences from 454 pyrosequencing data using Shannon's index (A) and Chao1 (B). The results of statistical analyses are provided in Table S1. Principle coordinate analysis of the unweighted UniFrac distance (C). Significant separation is present with respect to the origin of the mouse samples (*adonis, r^2^* = 0.15, *p* = 0.002).

To characterize the diversity of bacterial communities within individual mouse lungs and between individuals, we performed alpha and beta diversity analyses, respectively. Two different measures of alpha diversity focusing on species richness (Chao1) and evenness (Shannon) revealed notable differences between mice of different origins (Figure 5A–B, Table S1). Interestingly, the microbiota from non-SPF C57BL6 mice showed the highest diversity and richness, displaying significantly higher values (*p*<0.001) than mice of other origins in nearly all comparisons (Table S1).

In order to analyze the overall differences in community composition and structure between mice, we assessed beta diversity using both phylogenetic (unweighted and weighted UniFrac) and taxon (OTU)-based (Jaccard and Bray-Curtis) distances (Figure 5C, Figure S4 in File S1). By analyzing the samples with respect to their origin (*i.e*. SPF, non-SPF, wild-derived, wild-caught) using the multivariate ANOVA procedure implemented in analysis of dissimilarity (‘*adonis*’), three out of four of these distances displayed significant separation (*adonis*, unweighted UniFrac, *r^2^* = 0.15, *p* = 0.002; weighted UniFrac *r^2^* = 0.14, *p* = 0.16; Jaccard, *r^2^* = 0.16, *p* = 0.008; Bray-Curtis, *r^2^* = 0.19, *p* = 0.003) despite similarity at the phylum level as shown in Figure 4.

### Bacterial load and localization

**Figure 6:**
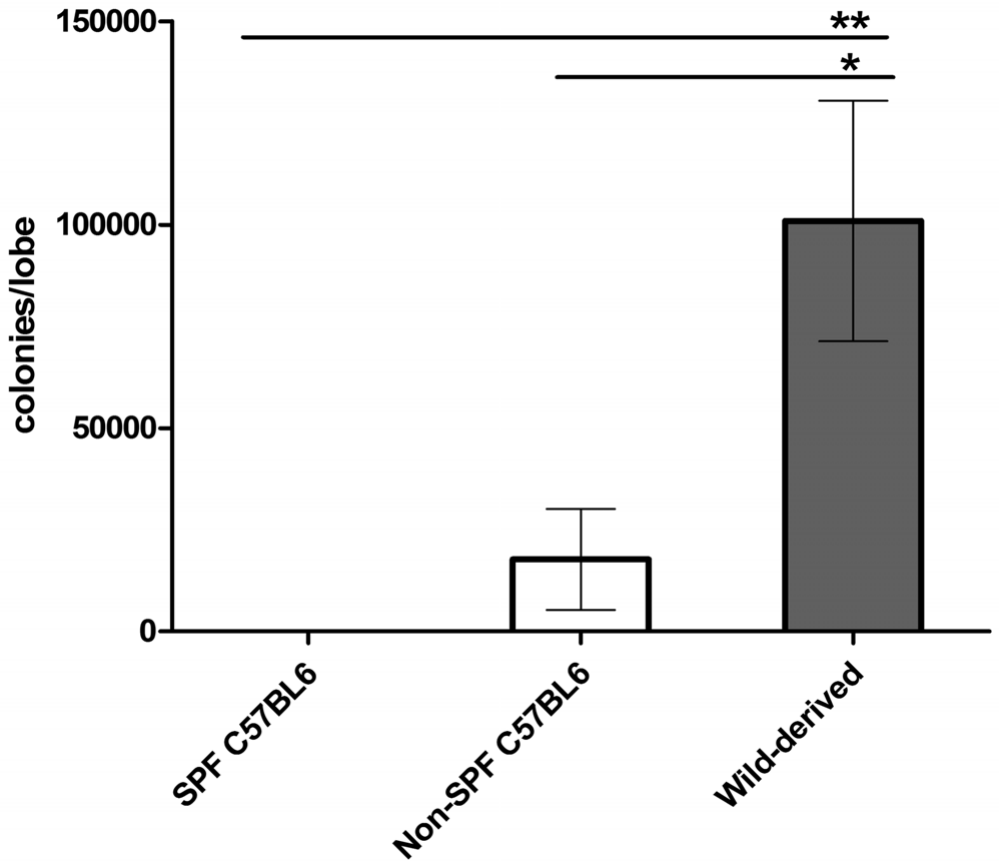
Enumeration of bacteria cultured from murine lungs. Colonies isolated from 6 non-SPF C57BL6 and 12 wild-derived mouse lungs by culture using 3 different culture conditions, i.e. BHI/aerobic, BAP/CO_2_ and BAP/anaerobic, were counted. Colony numbers were back-calculated as the colony forming units per lung lobe. No colonies were isolated from SPF-raised C57BL/6 mice. Two tailed Student's t-test followed by Mann Whitney test. Data shown are mean ± SEM.

To quantify cultivatable lung microbiota between mice of different origins, colony numbers of cultivatable bacteria were counted on each culture plate used for bacterial isolation. Numbers were recalculated to achieve the bacterial numbers per lung lobe. As shown in Figure 6, significantly lower numbers of bacteria were isolated from non-SPF C57BL6 than from wild-derived mice, although both were bred under comparable conditions within the same facility. The various types of colonies isolated from non-SPF lungs as depicted in Figure S2 in File S1 show that the differences in bacterial numbers were not due to overrepresentation of just one species. These results are interesting in light of the significantly higher richness as given by alpha diversity analyses in non-SPF C57BL6 compared to wild-derived mice (Figure 5), but indicate that animals raised in a microbe-poor environment (SPF) are prone to become associated with lower numbers but a wider variety of microbial settlers as compared to mice raised in a natural microbe-rich environment (Figure 5 and 6).

**Figure 7:**
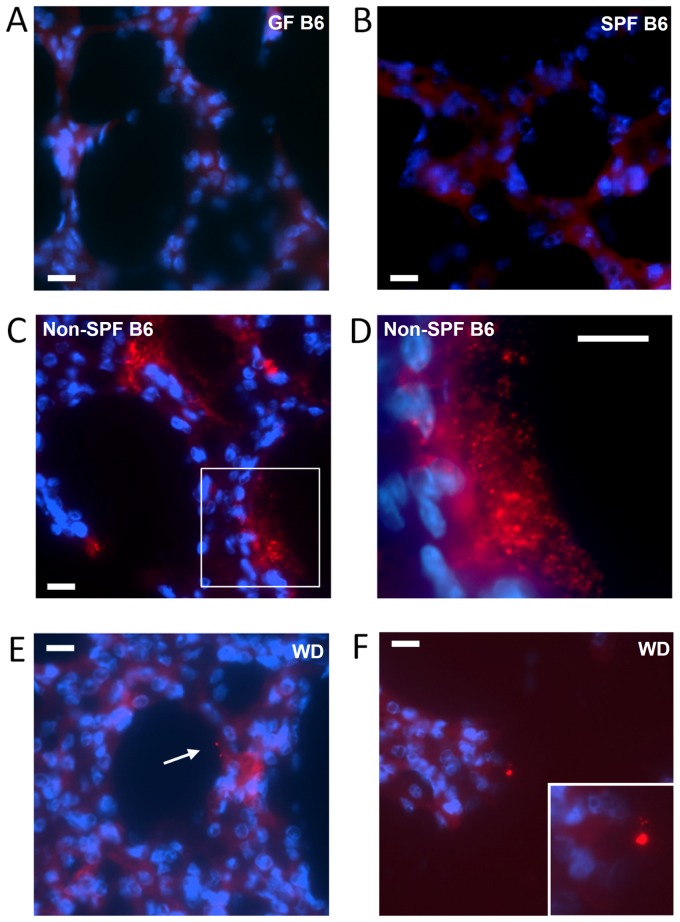
FISH of murine lungs. Lung sections from germ free C57BL/6 (A), SPF C57BL/6 (B), non-SPF C57BL/6 (C and D, higher magnification of the area in the white box in C), and wild-derived mice (E and F) were analyzed by FISH. Nuclei were stained with DAPI (blue). Bacteria were identified by hybridization with the broad-range bacterial probe EUB338 (red). White bars indicate 10 µm.

To determine the intrapulmonary localization of the murine microbiota, we employed FISH by using the eubacterial universal probe EUB338 as well as the EUB338 I, II, III mixture probe. We were unable to detect FISH signals in lung tissue sections from GF (Figure 7A) as well as SPF mice (Figure 7B). In contrast, positive FISH signals were detected in lung tissue sections from non-SPF C57BL/6 (Figure 7C-D; Figure S5Bc in File S1) as well as wild-derived mice (Figure 7E–F; Figure S5Bd–e in File S1). The bacterial communities detected by FISH varied with respect to their quantity from single cell-like signals to larger aggregates and biofilm-like formations. As shown in Figure 7D, FISH signals revealed bacterial aggregations of various sizes in close vicinity to and lining along the alveolar epithelium. However, consecutive sections stained by H&E for histological analysis did not reveal any obvious signs of inflammation (Figure S5A in File S1).

### Determination of lung histology by bacterial colonization

**Figure 8:**
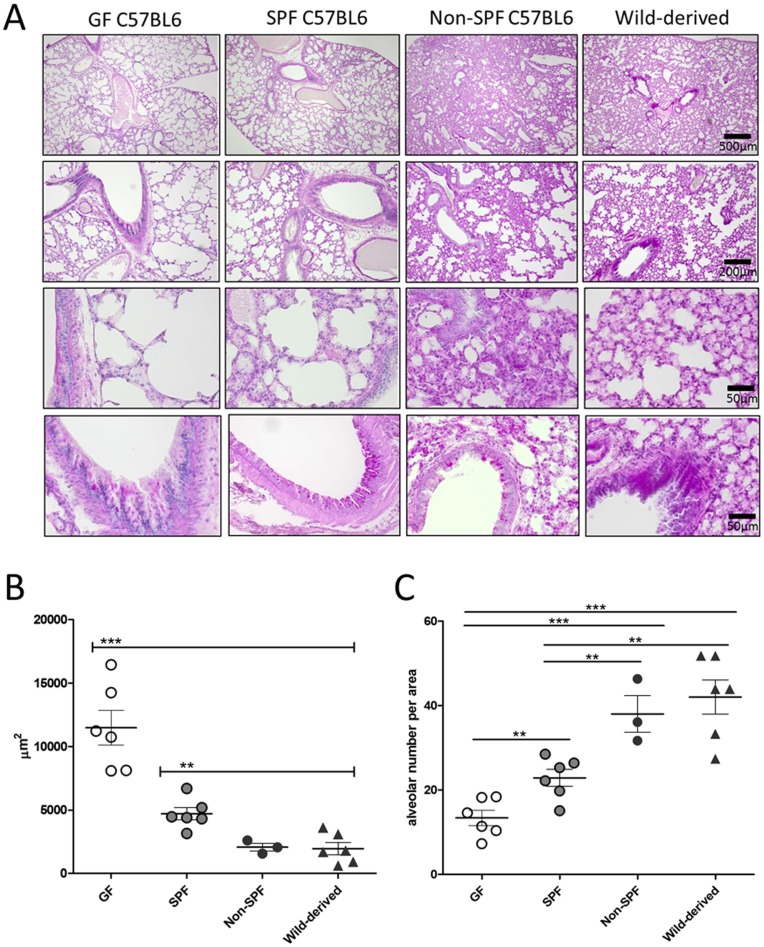
Lung microbiota influences alveolar morphology and mucus production. A. Histology of murine lungs from GF, SPF, non-SPF C57BL6 or wild-derived mice. Sections were stained with periodic acid-Schiff (PAS) and analyzed by light microscopy. Black bars indicate the magnification used (500 µm = 40X, 200 µm = 100X, 50 µm = 400X). Pictures show representative sections of 3–6 mice analyzed per group. B–C) Alveolar size as calculated from area measurements and the number of alveolae per microscopical field (400X magnification). Each dot represents the mean of either the area (µm^2^) from 10 alveolae (B) or the number of alveolae from 10 fields per section (C). Results from three to six mice per group are shown. Results are expressed as mean ± SEM, two tailed Student's t-test followed by Mann Whitney test.

Histological analyses revealed that the lung morphology between GF, SPF and non-SPF mice, which were of the same C57BL6 genetic background and age, was strikingly different. Less PAS positive staining of the alveolar region in both, GF and SPF C57BL/6 lungs when compared to non-SPF ones, indicated reduced mucus production in absence or low abundance of bacteria (Figure 8A). Diameters of alveolae were significantly smaller in non-SPF C57BL6 as well as wild-derived mice, with significantly more alveolae per lung area when compared to GF and SPF C57BL/6 ones (Figure 8B, C). Equal amounts of mucus were found in all mouse groups in the bronchial region. These observations indicate that the lung microbiota influences lung morphology and function.

**Figure 9:**
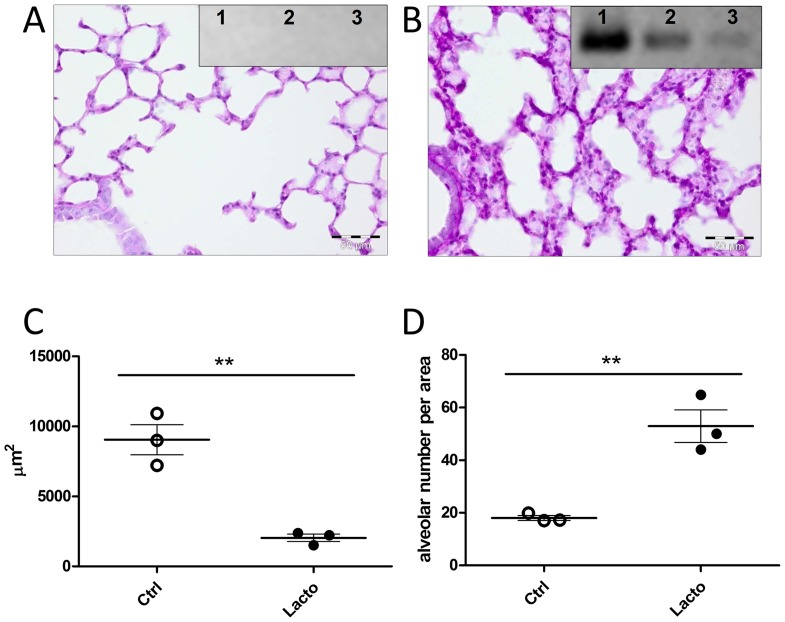
Alterations in lung histology upon transplantation of lung microbiota-derived Lactobacilli into germ-free mouse lungs. (A, B) Lungs from germ-free mice (A) or those inoculated with two *Lactobacillus* spp. isolates (B) were stained with PAS. Inserts depict agarose gel electrophoresis images of *Lactobacillus* spp.-specific PCR products (341bp) from 3 mice of each group. Pictures show representative sections from 1 out of 3 mice per group out of two similar experiments. (C, D) Alveolar size was calculated from area measurements (C) and the number of alveolae per microscopical field (D; 400X magnification). Each dot represents the mean of either the area (µm^2^) from 10 alveolae (C) or the number of alveolae from 10 fields per section (D). Results from three mice per group are shown. Non-treated control mice are termed ‘Ctrl’, and *Lactobacilli spp.* colonized ones ‘Lacto’. Results are expressed as mean ± SEM, two tailed Student's t-test followed by Mann Whitney test.

In order to confirm that bacterial colonization is responsible for the observed differences in lung architecture, GF mice were intranasally inoculated with selected bacterial isolates. Successful colonization of GF mice by *Lactobacillus* spp. in the lung was confirmed by genus-specific PCR (Figure 9). Compared to GF lungs, colonization with *Lactobacillus* spp. induced changes in the alveolar architecture, which was comparable to the lung morphology observed in non-SPF C57BL6 and wild-derived mice. Lungs colonized with lactobacilli showed enhanced mucus production as seen by PAS staining as well as significant differences in alveolar diameter and numbers when compared to GF lungs (compare Figure 9B to Figure 8A).

## Discussion

In contrast to the upper RT and other barrier epithelia exposed to the environment such as the gut, skin and vagina, the lower RT of a healthy human has been traditionally considered free of microorganisms. Recent analyses in humans, however, detected bacterial species in samples from the lower RT, suggesting the existence of a resident microbiota in the mammalian lung.

These studies suggested that, in contrast to the inhabitants of other epithelial surfaces, the microbiota of human lungs is of lower complexity. Bacterial species identified in BAL fluid from healthy donors included anaerobes, such as *Prevotella*, *Veillonella*, *Propionibacterium* [7], [25], indicating anaerobes as common members of the lower RT microbiota. It has also been suggested that these anaerobes may have the potential to become pathogenic under certain micro-ecological conditions, such as those found in CF patients. Together with *Prevotella* and *Veillonella* spp., Proteobacteria including *Haemophilus* spp. and *Neisseria* spp. were identified in bronchoscopy specimens from asthma patients [8]. *Pseudomonas* spp. represented the most dominant bacterial genus in BAL and lung explants of COPD patients [10]. Sterile sampling of human lower airway specimens is however a difficult issue as expectorated sputum and BAL fluid can easily become contaminated with bacterial residents of the upper airways such as trachea, oropharynx and sinuses [14], [26].

Our approach to analyze specimens from murine lungs excised and dissected under sterile conditions avoided upper respiratory tract contamination. In contrast to lungs from GF mice, those from mice raised under conventional or SPF conditions as well as those caught in the natural environment contained bacterial 16S rRNA. Taken together, cultivation as well as genetic identification by 16S rRNA sequencing allowed us to determine the lung microbiota of *Mus musculus*. The most dominant lung phyla found were Proteobacteria (64%), Firmicutes (20%), Actinobacteria (6%), and Bacteroidetes (4%), which shows a similar phylum distribution as previously reported for lung tissue microbiota from Balb/c mice [13]. Pasteurellaceae (Gammaproteobacteria) were found in wild mice derived from the environment, by both 454 sequencing as well as cultivation (Figure 3). *P. pneumotropica* is a ubiquitous bacterium that is frequently isolated from the upper respiratory tract of laboratory rodents including mice, rats, hamsters, and guinea pigs raised under non-SPF conditions [27]. Another member of the Pasteurellaceae, *Actinobacillus muris* can also colonize the oral cavity of mice and has been considered a commensal, since its presence could not be associated with pathological alterations [28]. Although quarantine measures to exclude carriers of *P. pneumotropica* from mouse facilities usually prevent colonization with this species, Pasteurallaceae (mainly *P. pneumotropica*) are the most commonly detected species in the laboratory mouse. Because of their frequent occurrence in laboratory mice all over the world, many animal facilities accept these microorganisms for pragmatic reasons [29]. Frequent pulmonary colonization by Pasteurellaceae indicates a close relationship between these species and the murine RT, similar to *Haemophilus influenzae* (which is also a member of Pasteurellaceae) commonly found in the upper airway of humans [26].

The majority of the lung isolates from each of the groups of mice included in our study contained the genera *Lactobacillus* (Figure 3, Table 2), which were primarily isolated under anaerobic conditions. Lactobacilli have proven probiotic activity upon oral treatment, which e.g. promotes resistance against enteropathogenic colonization, viral infection, and allergy [30], [31]. The natural habitat of lactobacilli ranges from fermenting dairy, meat and plant material to oral cavities and the genital and gastrointestinal tracts of humans and animals [32]. Therefore, it is not surprising to find lactobacilli in murine lungs. Further characterization of *Lactobacillus* lung-derived isolates and their function in airway homeostasis and immunity may reveal additional probiotic roles in respiratory diseases. Lactococci have been associated with lower incidence rates of atopic diseases in children who grew up in close association with certain farm animals [33].

Using RNA samples instead of DNA has the advantage of primarily identifying metabolically active bacteria, whereas according to Pezzulo et al, the majority of bacterial DNA from pulmonary material represents dead bacteria [12]. Overall, the Betaproteobacterium, *Ralstonia* spp., represents the most abundant bacterial genus as revealed by non-culture based analysis (Figure 1, 3). Since *Ralstonia* spp. is a frequent contaminant in DNA extraction procedures [34], we confirmed that its presence was not due to contamination during the experimental procedure as *Ralstonia*-specific PCR products were absent from GF lungs (Figure 2). Similarly, *Ralstonia* species have been isolated not only from the environment, but also from the upper RT of healthy humans [35].

Together, Firmicutes and Bacteroidetes represent 90–99% of the intestinal microbiota in humans and mice [36], but make up approximately 20% of the murine lung microbiota as determined by 454 sequencing (Figure 1). Due to their anaerobic metabolism, Bacteroidetes species may find only limited niches in the healthy lung. *Bacteroides* is a widely studied genus with respect to the immune function of the gut microbiota [1], [37]–[39], and represent 10–40% and 20% of the Bacteroidetes phylum identified in lungs from wild and non-SPF C57BL/6 mice, respectively, indicating a putative function for this genus also in pulmonary immune homeostasis. Notably, *Bacteroides* spp. were absent from the lungs of SPF mice (Figure S3D in File S1). Interestingly, *Sphingomonas* dominated the lung microbiota of C57BL6 mice from both SPF and non-SPF environments. *Sphingomonas* is a Gram-negative but lipopolysaccharide (LPS)-free bacterium, which produce glycosphingolipids, glycolipid antigens recognized by invariant natural killer T (iNKT) cells [40], [41]. As a commensal, *Sphingomonas* has been shown to contribute to immune homeostasis of iNKT cells [42].

Our analysis also revealed quantitative and qualitative changes in the lung microbiota between mice raised under SPF but transferred to conditions closer to the natural environment vs. those raised and kept under natural conditions. In the first group of mice, the number of sequences representing *Corynebacteriaceae* (phylum Actinobacteria; Figure S3A in File S1) and *Bacteroidetes* was high (Figure S3B in File S1) and the number of those representing *Chitinophagaceae* (Figure S3B in File S1) and *Moraxellaceae* (Figure S3E in File S1) was low. More importantly, non-SPF C57BL/6 mice displayed the highest species diversity of all mouse groups studied, even when mice caught in the wild were considered (Figure 5B). According to the “Hygiene hypothesis”, this result suggests that mice raised under “clean” SPF conditions lack the exposure to a variety of “natural” commensal species, which may cause insufficiently developed lung immunity rendering those mice more vulnerable to colonization by a wider variety of bacterial species which may be maintained in the population [43].

The influence of a different environment is also evident in wild mice derived from a mouse colony the founders thereof originated from the natural environment before transferred to an indoor animal facility for breeding. Lungs of these wild-derived mice contained higher numbers of sequences from *Corynebacteriaceae* and *Microbacteriaceae* but less sequences from Actinobacteria and Firmicutes (Figure 4, Figure S3 in File S1). Although *Helicobacter* spp. was abundant in wild mice caught in the environment, this genus was less often found in wild-derived mice housed indoors (Figure 4). However, apart from these differences, the overall differences in diversity observed between wild-caught and wild-derived mice were not as a dramatic as those between SPF and non-SPF mice (Figure 5B). This phenomenon could be due to colonization resistance, i.e. once an intact microbiota is formed additional bacterial species only mildly influence the epithelial ecosystem, as observed in an animal model for enteropathogenic bacteria colonization [30]. Similar to the lung, the bacterial microbiota of the stomach is of relatively low complexity, which has been suggested not only to be determined by niche-specific factors but also by stochastic colonization from the upstream alimentary tract [44]. Thus, the lung could also in part be comprised of stochastic colonizers from the upper RT. However, it should be noted that the colonies identified by FISH indicate specific niches in the lung where bacteria can build biofilm-like communities (Figure 7C–D). Interestingly, the largest bacterial aggregations were found in non-SPF C57BL/6 lungs, which also displayed the highest bacterial species diversity (Figure 5).

Finally and most importantly, the differences in alveolar morphology and mucus production (Figure 8) in relation to the abundance of bacteria in the lung are striking observations. This unexpected finding was confirmed by associating germfree mice with two *Lactobacillus* isolates from the murine lung (Figure 9), which lead to similar alterations in alveolar numbers, size and mucus production as observed in non-SPF lungs. As age and genetic background may influence lung architecture, we should note that the differences in alveolar size and counts were observed between GF, SPF and non-SPF mice of the same C57BL/6 background and age. Taken together, these results strongly suggest that bacteria influence lung development and barrier functions. Future studies will focus on the stability and localization of bacterial colonization and its influence on pulmonary immune responses. Effects of microbiota on organ development have recently been revealed for a number of tissues including the intestinal crypts, lymphoid tissue and the blood-brain barrier [45], [46]. Instruction of innate immune responses including epithelial defense function is also triggered by the presence of microbiota [31], [47]. Thus, enhanced alveolar mucus production could be due to a direct effect of alveolar settlers, whereas the bronchial mucus production is rather influenced by inhaled microbes from the upper respiratory tract. However, whether the increase in numbers of alveolae and corresponding reduction in alveolar size requires an indigenous lung community, or general microbial settlement at other sites such as the gut, will require further colonization experiments.

Our description of a murine lung microbiota by both cultivation and genetic methods along with its dependency on the animal's environmental conditions opens the path for functional studies on the role of lung commensals in airway morphogenesis, epithelial homeostasis and immunity. In particular cultivatable bacteria can be systematically investigated for their colonization and growth characteristics on lung epithelia, their influence on antibiotic activity, lung infection and inflammation. This may ultimately enable the discovery of novel immune modulating or probiotic properties for prophylactic or therapeutic measures against airway disorders.

## Supporting Information

File S1 **Figure S1. Phylogenetic tree of isolates and clones from mouse lungs determined by 16S rRNA gene sequences**. The Neighbor-Joining method implemented in MEGA was applied to 128 taxa from colony isolates and 103 taxa from cloned library in addition to 33 representative bacterial sequences identified by BLAST and RDP. Branches corresponding to partitions reproduced in less than 50% bootstrap replicates are collapsed. More than 99% identical reads were given by number with isolates in blue and clones in red. In addition, strain names with green background shows deposited strains in DSMZ. **Figure S2. Examples of colonies cultured from mouse lungs under different culture conditions.** Homogenized lung material was plated onto Blood agar (BAP)/chocolate agar plate (CH) in 5% CO_2_, Luria Burtani (LB)/Brain Heart Infusion (BHI) and BAP under aerobic or anaerobic conditions, respectively. **Figure S3. Differences at the genus or family level in each phylum between mouse origins.** **A.** Actinobacteria, **B.** Bacteroidetes, **C.** Firmicutes, **D.** Alphaproteobacteria, **E.** Gammaproteobacteria. Constructed data within each phylum was extracted from 454 pyrosequencing analysis shown in Figure 4. **Figure S4**. Beta analysis for Bray-curtis distance measurement based on the taxon abundance revealed significant community differences among individuals from different categories (Adonis: R^2^ = 0.18, *p* = 0.003). The Jaccard distance measurement based on presence or absence of taxon also revealed similar trend (Adonis: R^2^ = 0.15, *p* = 0.008). Similar results were obtained when the phylogenetic-based measurements on taxon abundance was included (i.e. Weighted UniFrac, Adonis: R^2^ = 0.13, *p* is not significant). **Figure S5. A. Histopathology of murine lungs.** Lung sections from germ-free C57BL/6 (a), SPF C57BL/6 (b), non-SPF C57BL/6 (c) and wild-derived mice (d and e) were analyzed by H&E staining. Histology reveals no obvious signs of inflammatory responses. **B. FISH of murine lungs.** Lung sections from germ-free C57BL/6 (a), SPF C57BL/6 (b), non-SPF C57BL/6 (c) and wild-derived mice (d and e) were analyzed using the EUB338 I, II and III mixture probe (red) [48] and subsequently stained with DAPI (blue) to depict nuclei. The pictures corresponded to the histological images shown in S5A. The control Non-EUB338 probe is shown in (f). (Magnification 100X).
(PPT)

Table S1 Significance of alpha diversity comparisons.
(DOC)
