# Supplementary material for: Environmentally Determined Differences in the Murine Lung Microbiota and Their Relation to Alveolar Architecture
Source: PLoS One. 2014 Dec 3;9(12):e113466. doi: 10.1371/journal.pone.0113466 (PMC4254600; doi:10.1371/journal.pone.0113466)
Supplement: File S1 — Figure S1. Phylogenetic tree of isolates and clones from mouse lungs determined by 16S rRNA gene sequences. The Neighbor-Joining method implemented in MEGA was applied to 128 taxa from colony isolates and 103 taxa from cloned library in addition to 33 representative bacterial sequences identified by BLAST and RDP. Branches corresponding to partitions reproduced in less than 50% bootstrap replicates are collapsed. More than 99% identical reads were given by number with isolates in blue and clones in red. In addition, strain names with green background shows deposited strains in DSMZ. Figure S2. Examples of colonies cultured from mouse lungs under different culture conditions. Homogenized lung material was plated onto Blood agar (BAP)/chocolate agar plate (CH) in 5% CO2, Luria Burtani (LB)/Brain Heart Infusion (BHI) and BAP under aerobic or anaerobic conditions, respectively. Figure S3. Differences at the genus or family level in each phylum between mouse origins. A. Actinobacteria, B. Bacteroidetes, C. Firmicutes, D. Alphaproteobacteria, E. Gammaproteobacteria. Constructed data within each phylum was extracted from 454 pyrosequencing analysis shown in Figure 4. Figure S4. Beta analysis for Bray-curtis distance measurement based on the taxon abundance revealed significant community differences among individuals from different categories (Adonis: R2 = 0.18, p = 0.003). The Jaccard distance measurement based on presence or absence of taxon also revealed similar trend (Adonis: R2 = 0.15, p = 0.008). Similar results were obtained when the phylogenetic-based measurements on taxon abundance was included (i.e. Weighted UniFrac, Adonis: R2 = 0.13, p is not significant). Figure S5. A. Histopathology of murine lungs. Lung sections from germ-free C57BL/6 (a), SPF C57BL/6 (b), non-SPF C57BL/6 (c) and wild-derived mice (d and e) were analyzed by H&E staining. Histology reveals no obvious signs of inflammatory responses. B. FISH of murine lungs. Lung sections from germ-free C57BL/6 [file pone.0113466.s001.ppt]

## Slide 1
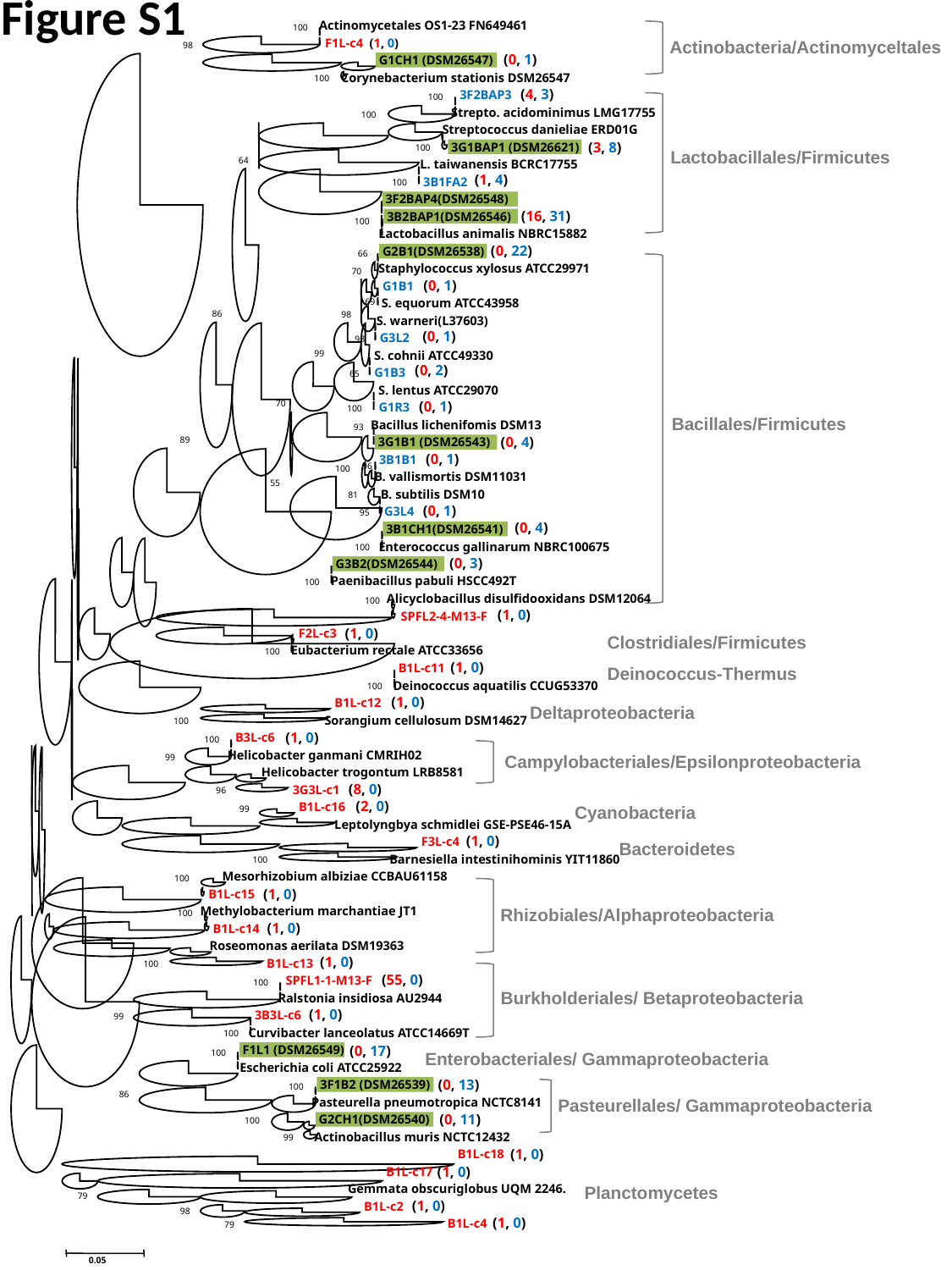

Figure S1
 Actinomycetales OS1-23 FN649461
 F1L-c4 (1, 0)
 G1CH1 (DSM26547)
 Corynebacterium stationis DSM26547
 3F2BAP3
 Strepto. acidominimus LMG17755
 Streptococcus danieliae ERD01G
 3G1BAP1 (DSM26621)
 L. taiwanensis BCRC17755
 3B1FA2
 3F2BAP4(DSM26548)
 3B2BAP1(DSM26546)
 Lactobacillus animalis NBRC15882
 G2B1(DSM26538)
 Staphylococcus xylosus ATCC29971
 G1B1
 S. equorum ATCC43958
 S. warneri(L37603)
 G3L2
 S. cohnii ATCC49330
 G1B3
 S. lentus ATCC29070
 G1R3
 Bacillus lichenifomis DSM13
 3G1B1 (DSM26543)
 3B1B1
 B. vallismortis DSM11031
 B. subtilis DSM10
 G3L4
 3B1CH1(DSM26541)
 Enterococcus gallinarum NBRC100675
 G3B2(DSM26544)
 Paenibacillus pabuli HSCC492T
 Alicyclobacillus disulfidooxidans DSM12064
 SPFL2-4-M13-F
 F2L-c3
 Eubacterium rectale ATCC33656
 B1L-c11
 Deinococcus aquatilis CCUG53370
 B1L-c12
 Sorangium cellulosum DSM14627
 B3L-c6
 Helicobacter ganmani CMRIH02
 Helicobacter trogontum LRB8581
 3G3L-c1
 B1L-c16
 Leptolyngbya schmidlei GSE-PSE46-15A
 F3L-c4
 Barnesiella intestinihominis YIT11860
 Mesorhizobium albiziae CCBAU61158
 B1L-c15
 Methylobacterium marchantiae JT1
 B1L-c14
 Roseomonas aerilata DSM19363
 B1L-c13
 SPFL1-1-M13-F
 Ralstonia insidiosa AU2944
 3B3L-c6
 Curvibacter lanceolatus ATCC14669T
 F1L1 (DSM26549)
 Escherichia coli ATCC25922
 3F1B2 (DSM26539)
 Pasteurella pneumotropica NCTC8141
 G2CH1(DSM26540)
 Actinobacillus muris NCTC12432
 B1L-c18
 B1L-c17
 Gemmata obscuriglobus UQM 2246.
100
98
100
100
100
100
64
100
100
66
70
69
86
98
98
99
65
70
100
93
89
96
100
55
81
95
100
100
100
100
100
100
100
99
96
99
100
100
100
100
100
99
100
100
100
86
100
99
79
 B1L-c2
98
 B1L-c4
79
0.05
Actinobacteria/Actinomyceltales
(0, 1)
(4, 3)
(3, 8)
Lactobacillales/Firmicutes
(1, 4)
(16, 31)
(0, 22)
(0, 1)
(0, 1)
(0, 2)
(0, 1)
Bacillales/Firmicutes
(0, 4)
(0, 1)
(0, 1)
(0, 4)
(0, 3)
(1, 0)
(1, 0)
Clostridiales/Firmicutes
(1, 0)
Deinococcus-Thermus
(1, 0)
Deltaproteobacteria
(1, 0)
Campylobacteriales/Epsilonproteobacteria
(8, 0)
(2, 0)
Cyanobacteria
(1, 0)
Bacteroidetes
(1, 0)
Rhizobiales/Alphaproteobacteria
(1, 0)
(1, 0)
(55, 0)
Burkholderiales/ Betaproteobacteria
(1, 0)
(0, 17)
Enterobacteriales/ Gammaproteobacteria
(0, 13)
Pasteurellales/ Gammaproteobacteria
(0, 11)
(1, 0)
(1, 0)
Planctomycetes
(1, 0)
(1, 0)

## Slide 2
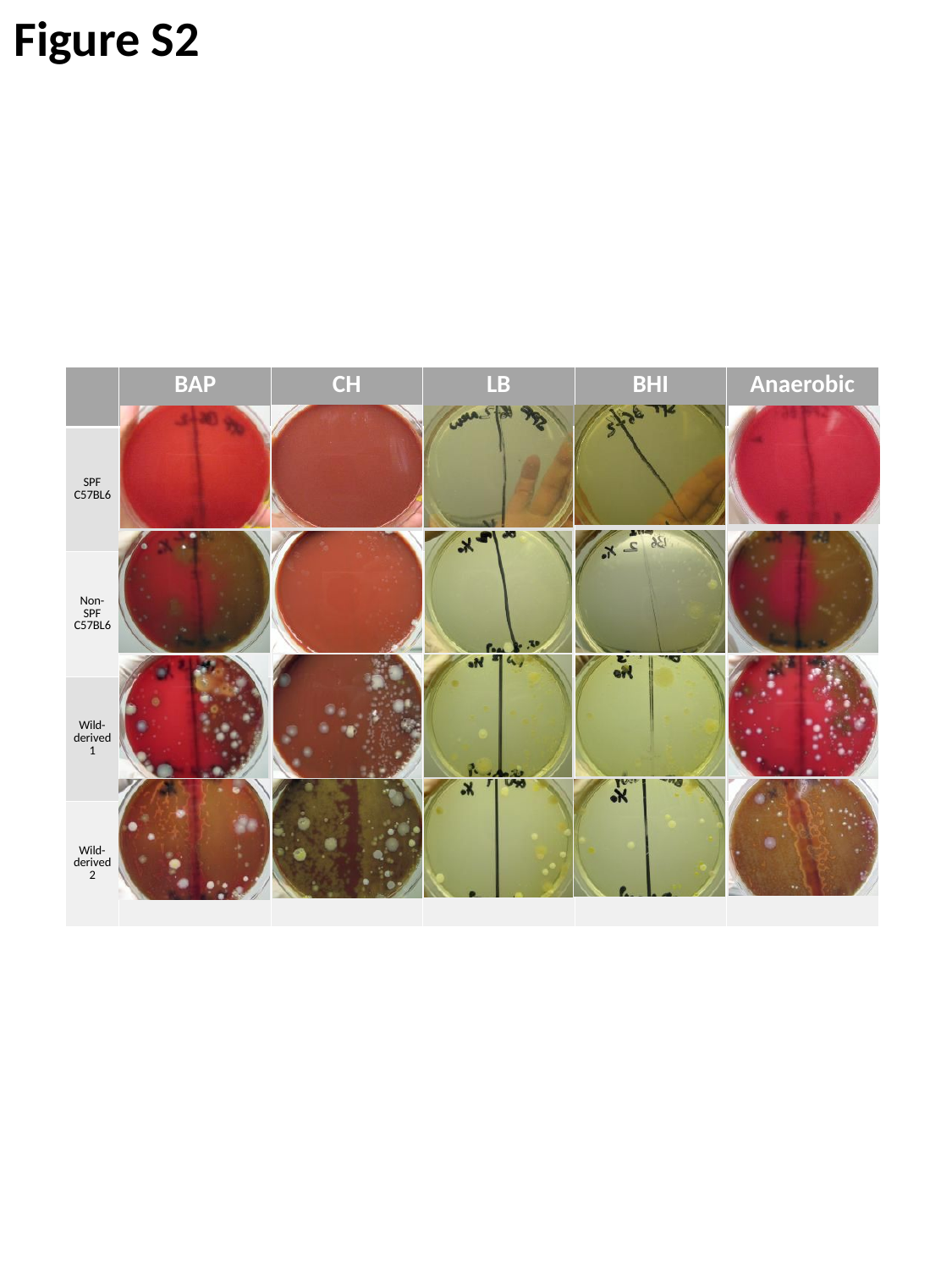

Figure S2
| | BAP | CH | LB | BHI | Anaerobic |
| --- | --- | --- | --- | --- | --- |
| SPF C57BL6 | | | | | |
| Non- SPF C57BL6 | | | | | |
| Wild- derived 1 | | | | | |
| Wild- derived 2 | | | | | |

## Slide 3
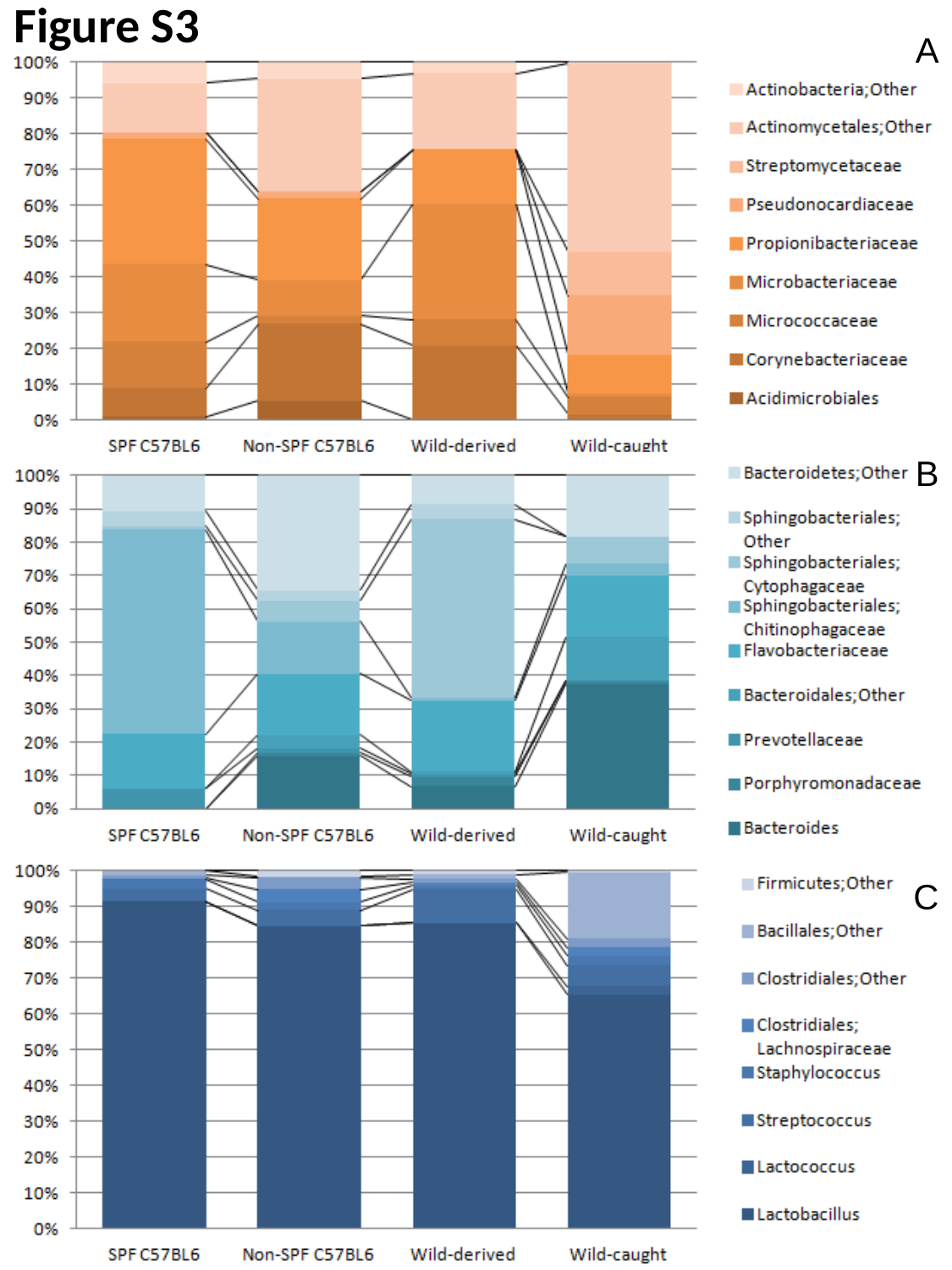

Figure S3
A
B
C

## Slide 4
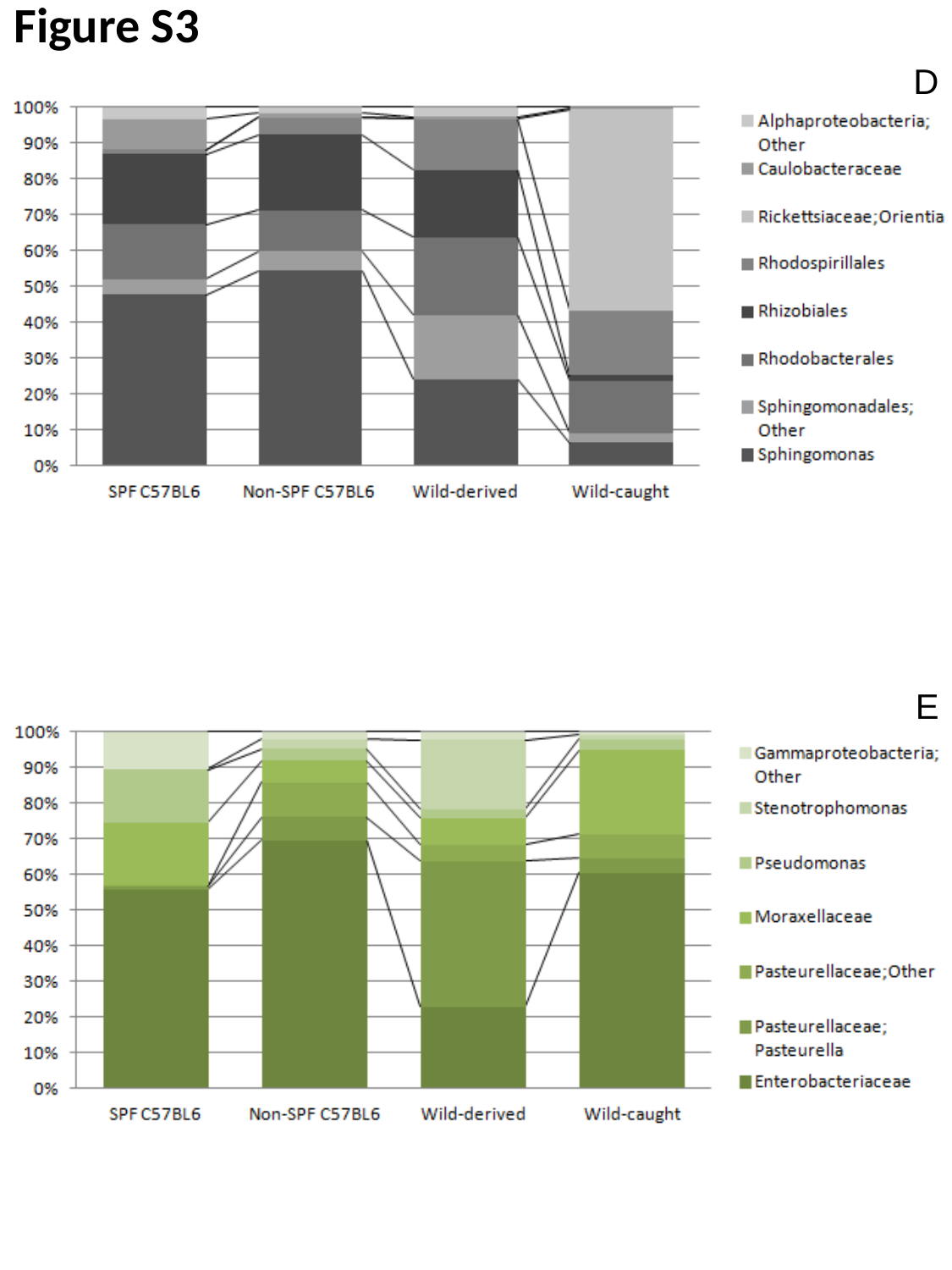

Figure S3
D
E

## Slide 5
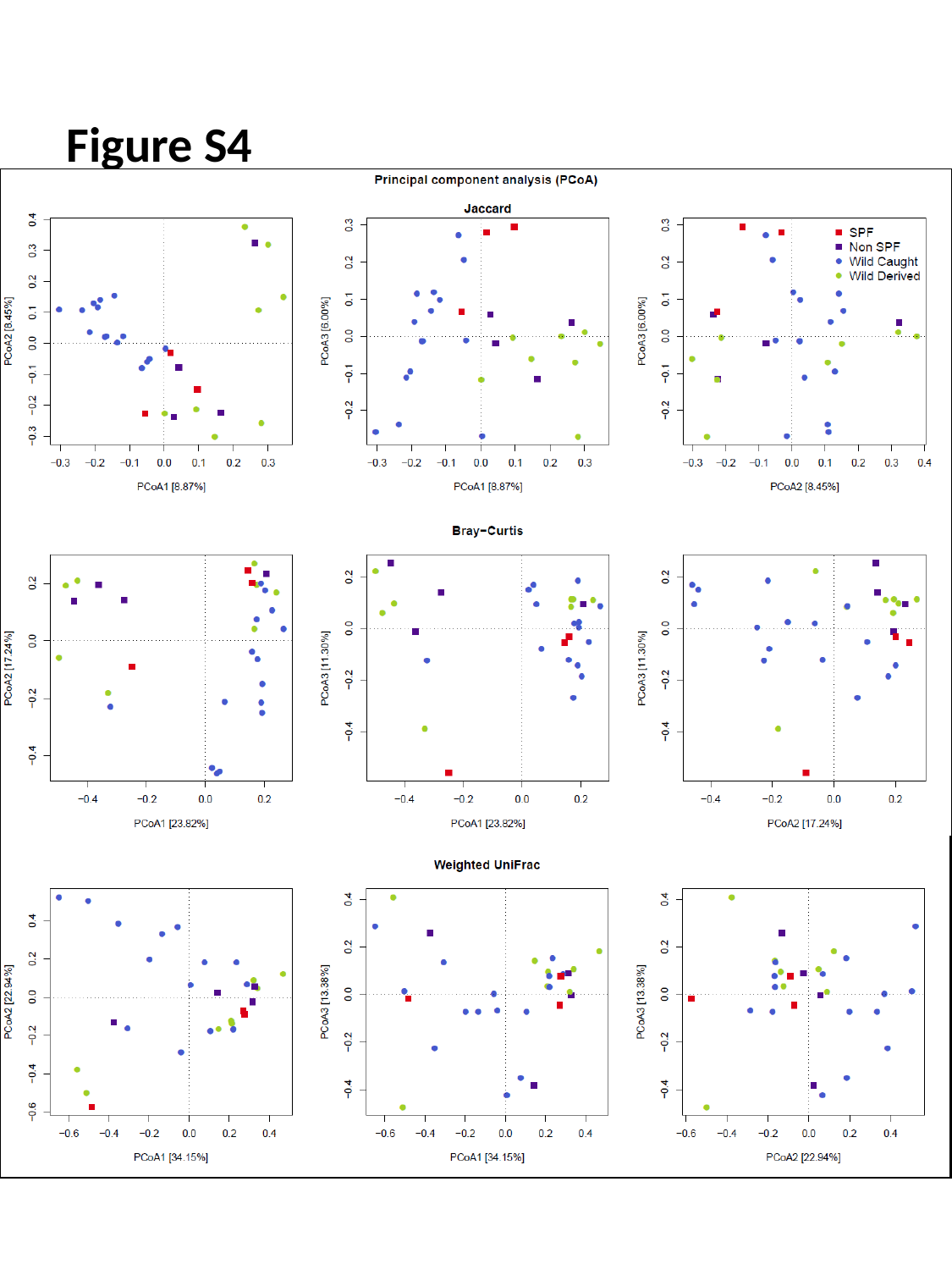

Figure S4

## Slide 6
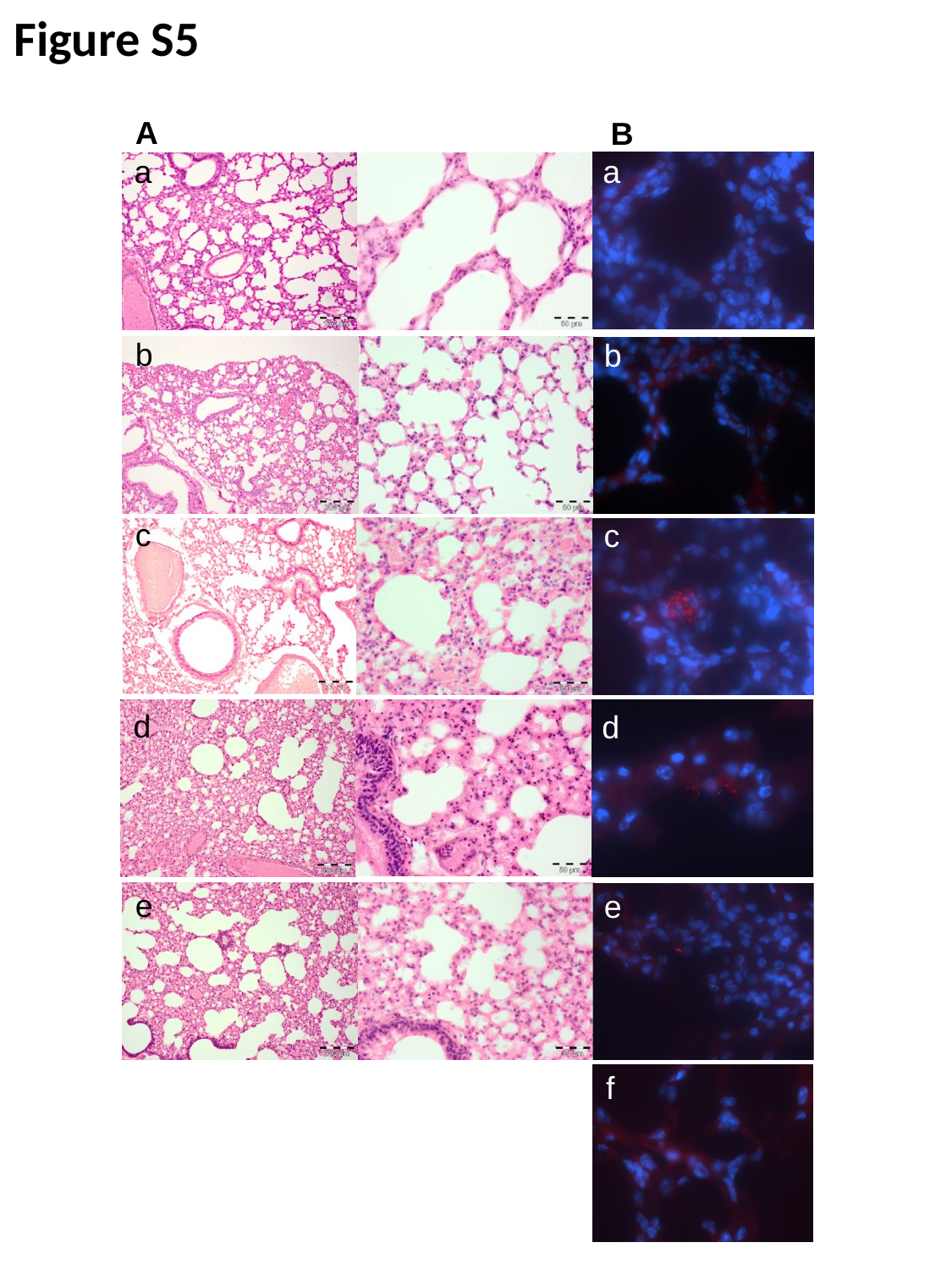

Figure S5
A
B
a
a
b
b
c
c
d
d
e
e
f
