# Supplementary material for: Environmentally Determined Differences in the Murine Lung Microbiota and Their Relation to Alveolar Architecture
Source: PLoS One. 2014 Dec 3;9(12):e113466. doi: 10.1371/journal.pone.0113466 (PMC4254600; doi:10.1371/journal.pone.0113466)
Supplement: Table S1 — Significance of alpha diversity comparisons. (DOC) [file pone.0113466.s002.doc]

**Supplemental Table S1.** Significance of alpha diversity comparisons was determined by the Wilcoxon rank sum test. Significant comparisons after adjusting *p* values for multiple testing are indicated in bold.

|  | Chao1 *p* value* | Shannon *p* value* |
| --- | --- | --- |
| Wild caught vs. SPF | **0.002** | **<0.001** |
| Wild caught vs. non SPF | **<0.001** | **<0.001** |
| Wild caught vs. Wild derived | 1 | **0.02** |
| Wild derived vs. non SPF | **<0.001** | **<0.001** |
| Wild derived vs. SPF | 0.17 | **<0.001** |
| SPF vs. non SPF | **0.006** | 0.12 |

*Adjusted for multiple testing using the method of Benjamini and Hochberg.

1. Benjamini Y, Hochberg Y (1995) Controlling the False Discovery Rate: A Practical and Powerful Approach to Multiple Testing. J R Stat Soc B57: 289-300.
